# A decision-making framework to maximise the evolutionary potential of populations - Genetic and genomic insights from the common midwife toad (*Alytes obstetricans*) at its range limits

**DOI:** 10.1038/s41437-024-00710-4

**Published:** 2024-09-02

**Authors:** Christopher D. Barratt, Kathleen Preißler, Pauline R. Jennert, Falk Eckhardt, Mirjam Nadjafzadeh, Sebastian Steinfartz

**Affiliations:** 1 German Centre for Integrative Biodiversity Research (iDiv) Halle-Jena-Leipzig, Puschstrasse 4, 04103 Leipzig, Germany; 2 University of Leipzig, Ritterstrasse 26, 04109 Leipzig, Germany; 3 Naturalis Biodiversity Center, Darwinweg 2, 2333 CR Leiden, The Netherlands; 4 Animal Breeding and Genomics, Wageningen University & Research, Droevendaalsesteeg 1, 6708 PB Wageningen, The Netherlands; 5 University of Leipzig, Institute of Biology, Molecular Evolution and Systematics of Animals, Talstrasse 33, 04103 Leipzig, Germany; 6 NABU (Nature and Biodiversity Conservation Union) Lower Saxony, Alleestrasse 36, 30167 Hannover, Germany

**Keywords:** Population genetics, Conservation biology, Molecular ecology

## Abstract

Anthropogenic habitat modification and climate change are fundamental drivers of biodiversity declines, reducing the evolutionary potential of species, particularly at their distributional limits. Supportive breeding or reintroductions of individuals are often made to replenish declining populations, sometimes informed by genetic analysis. However, most approaches utilised (i.e. single locus markers) do not have the resolution to account for local adaptation to environmental conditions, a crucial aspect to consider when selecting donor and recipient populations. Here, we incorporate genetic (microsatellite) and genome-wide SNP (ddRAD-seq) markers, accounting for both neutral and putative adaptive genetic diversity, to inform the conservation management of the threatened common midwife toad, *Alytes obstetricans* at the northern and eastern edges of its range in Europe. We find geographically structured populations (*n* = 4), weak genetic differentiation and fairly consistent levels of genetic diversity across localities (observed heterozygosity and allelic richness). Categorising individuals based on putatively adaptive regions of the genome showed that the majority of localities are not strongly locally adapted. However, several localities present high numbers of private alleles in tandem with local adaptation to warmer conditions and rough topography. Combining genetic diversity and local adaptations with estimates of migration rates, we develop a decision-making framework for selecting donor and recipient populations which maximises the geographic dispersal of neutral and putatively adaptive genetic diversity. Our framework is generally applicable to any species, but especially to amphibians, so armed with this information, conservationists may avoid the reintroduction of unsuitable/maladapted individuals to new sites and increase the evolutionary potential of populations within species.

## Introduction

Globally, species are threatened by anthropogenic pressure and rapid environmental change resulting in the loss and fragmentation of habitats (IPBES 2019). Together, geographically distinct localities and populations constitute a species’ gene pool. It is broadly accepted that the more diverse a species’ gene pool is, the higher its evolutionary potential—i.e. the ability to adapt to new and changing biotic as well as abiotic environmental conditions—and accordingly its resilience against natural and anthropogenically caused environmental challenges (IPCC 2014). Incorporating evolutionary information to understand population structure and quantify genetic diversity is a fundamental principle in the field of conservation genetics (Frankham et al. 2002, 2019). A better understanding of geographic variation in genetic diversity within species facilitates targeted conservation efforts to preserve localities and populations of particular importance, and to manage gene flow between populations to ensure that genetic diversity is not eroded over time, thus maintaining the species’ overall genetic diversity and evolutionary potential.

Through the use of genome-wide SNPs previously intractable questions about genetic diversity in non-model organisms can be addressed (Andrews et al. 2016). Excitingly, for our understanding of evolutionary potential, genome-wide SNPs enable us to look beyond ‘neutral’ genetic diversity (Teixeira and Huber 2021), to provide insights into the ‘adaptive capacity’ of a species (Foden et al. 2019). By identifying regions of the genome that may be under putative selection and quantifying the distribution of adaptive alleles across conspecific populations using GEA (Genotype-Environment Association) approaches (Capblanq and Forester 2021), we are able to assess local adaptation to changing environmental conditions. Assessing both neutral and putatively adaptive genetic diversity together, we are able to gain a clearer understanding of the overall evolutionary potential of species and their conspecific populations, and to develop decision making frameworks for the management of populations that account for the novel insights provided by genomic data (e.g. Bossu et al. 2023).

Due to their high sensitivity to environmental and anthropogenic change (Wake and Vredenburg 2008), and with 35% of species listed as “threatened” by the IUCN (2022), amphibians are an ideal system to investigate the spatial variability of evolutionary potential, and how to maintain it within species (Forester et al. 2022). In this work, we focus on *Alytes obstetricans*, the common midwife toad, at its northern and eastern range limits in Europe as a case study to develop an integrative decision-making framework to maximise evolutionary potential in different populations. The genetic characterisation of the species southern and western range has been thoroughly explored before (Gonçalves et al. 2015; Maia-Carvalho et al. 2014, 2018; Dufresnes and Martínez-Solano 2020; Ambu et al. 2023), while comparatively less attention has been paid to its northern and eastern range. *Alytes obstetricans* is the most widely distributed member of its genus (which consists of five European species), present across western and central Europe (northern Spain and Portugal, France, Belgium, Luxembourg, the Netherlands, Switzerland and Germany) (IUCN 2022). As with its congeners, *A. obstetricans* exhibits a special reproductive mode, in which the male carries the fertilised eggs until the larvae hatch to be deposited in the aquatic habitat where they develop until metamorphosis. The species inhabits a wide range of open to semi-open, structurally rich landscapes and predominantly vegetation-poor areas, in which sun-exposed hiding places are common; often on hillside embankments as well as rocky areas (Speybroeck et al. 2010). The aquatic habitats for reproduction are often found in the immediate vicinity to the terrestrial habitats and represent a broad spectrum of water bodies, such as small to medium-sized ponds or quarry waters. It is important that the breeding waters are mainly permanently water-bearing as some of the larvae are deposited relatively late (July or August) and must hibernate in the water bodies until the following year. Environmental threats to midwife toads comprise habitat loss and fragmentation which have been the main causes of their rapid decline in many areas of their range (Barrios et al. 2012). Suitable areas for shelter, foraging and mating are lost or degraded where landscape structures such as sun-exposed slopes, field margins, stone walls and stone piles are removed. The loss of breeding waters occurred mainly due to groundwater lowering because of intensified agriculture, infilling, pollution by garbage, fertilisers and other environmental toxins as well as fish stocking. On top of this, they are further threatened by increasing and prolonged periods of drought due to climate change, which favour the drying up of spawning waters early in the year. Finally, the fragmentation of metapopulations and the resulting lack of genetic exchange could pose a serious problem for the long-term viability of this species. As a consequence, populations of *A. obstetricans* are protected throughout the European Union according to the Habitats Directive (annex IV) and belong to the “specially and strictly protected species” according to the German Federal Nature Conservation Act. Germany bears a special responsibility for *A. obstetricans* since it marks the species’ northern and eastern distribution limits, with small, isolated and declining populations as a result of habitat loss and fragmentation (Grossenbacher and Zumbach 2003). Consequently, *A. obstetricans* was recently uplisted to “Endangered” on the German Red List (Scheidt 2020).

**Fig. 1:**
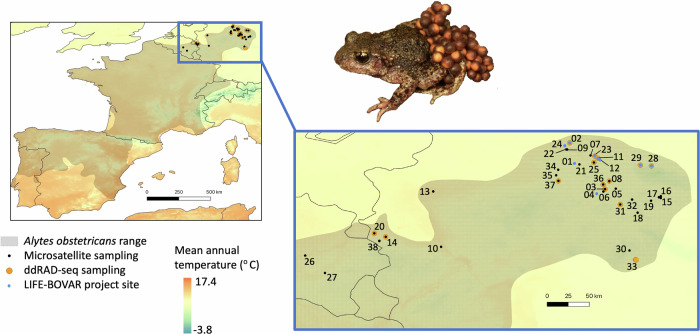
Sampling the northern and eastern range limits of *Alytes obstetricans.* IUCN range map shows the whole range of the species in Europe, with our sampling region highlighted (blue bounding box). Sampling locality information (numbers 01-38 and their geographic coordinates) are shown in Table 1. A photo of a male *A. obstetricans* carrying eggs is shown (Credit: Miguel Vences).

Habitat modification and loss, together with other factors (such as disease) is influencing the genetic diversity of amphibians across the earth due to the combined effects of population declines, reduced effective population sizes and increased genetic drift and inbreeding (Allentoft and O’Brien 2010). Genetic diversity loss has often been attributed with a direct negative effect on fitness, and the enhancement of genetic diversity as a general management strategy has been promoted to increase resilience to current threats (Pröhl and Rodríguez 2023). However, most previous genetic diversity estimates are based on neutral genetic markers, and major knowledge gaps exist about whether individuals used as sources for translocations or genetic rescue are suitable for enhancing population viability. For example, the absence of information about local adaptations may mean that individuals being introduced to populations to bolster genetic diversity are potentially maladapted to the local environmental conditions and may not necessarily increase population viability. In order to counteract the negative population trend of *A. obstericans* populations in Germany and to sustain the viability and the adaptive potential of their populations, an eight year EU funded project, LIFE BOVAR, implemented by the NABU (Nature and Biodiversity Conservation Union) of Lower Saxony contributes to the restoration of the original range of several target species including *A. obstetricans* by supportive breeding and reintroduction in selected areas. This is implemented according to the IUCN Guidelines (IUCN/SSC 2013). Key to the decision-making process of conservation interventions are knowledge of dispersal routes as well as the genetic structure, diversity and local adaptations of potential source populations, used as a basis for the reintroduction of populations, and improving metapopulation connectivity. Accordingly, we identified general population structure and genetic diversity of *A. obstetricans* localities across their range limits with microsatellite markers. In addition, we selected a subset of individuals to create a genomic dataset using Double Digest Restriction-site Associated DNA-sequencing (ddRAD-seq) to enable the investigation of evolutionary potential and dispersal capabilities across sampled localities using both neutral and putatively adaptive molecular markers. We used these data, as well as detailed knowledge on habitat suitability and availability across management sites to inform our decision-making process for selecting suitable donor and recipient populations to also include adaptive diversity in conservation planning, in line with approaches by Flanagan et al. (2018). Though our framework is addressing the midwife toad *A. obstetricans* mainly across our LIFE-BOVAR project management sites and surrounding localities in the Eastern part of its range in Lower Saxony (Fig. 1 and Table 2), we believe that it can be in principle also applied to other species and systems to develop decision-making frameworks maximising evolutionary potential of threatened species in the in wild.

## Materials and methods

### Sampling and DNA extraction

In 2018–2020, a total of 652 individual common midwife toads (*Alytes obstetricans*) were sampled via buccal swabbing (adults) and fin seam-clipping (larvae) at 36 sites across Germany and at two sites in Belgium (Table 1, which includes sample life stage information per sampling locality). This minimally invasive sampling did not result in any impairment of the sampled individuals and complied with relevant EU laws for the protection of animals used for scientific purposes (2010/63/EU). Samples were stored in centrifuge tubes with (fin seam tissue) or without (swabs) 96% ethanol. Genomic DNA was extracted from swabs using the DNeasy Blood & Tissue Kit (Qiagen) and from tissue using a two-phased phenol chloroform isoamyl alcohol extraction protocol (Supplementary Material text S1, modified from Gilbert et al. 2007). DNA isolates were stored at −20 °C. Though logistical and financial limitations meant that we were unable to sample every single known locality in our study region for *A. obstetricans*, our sampling regime provides high spatial resolution data for a total of 38 localities across the eastern and northern range limits of *A. obstetricans* that are undergoing declines (Fig. 1).

**Table 1:** Sampling information for all *Alytes obstetricans* individuals included in this study.

Sampling localities	Latitude	Longitude	Total n samples	Microsatellite n samples	ddRAD-seq *n* samples	LS
Luegde (01)*	51.93819	9.281583	22	19		L
Obernkirchen (02)*	52.26517	9.208136	20	17	5	L
Helenenwiese (03)	51.53074	9.768711	20	20	4	L
Ballertasche (04)*	51.45833	9.635669	6	6		A
Goettingen (05)	51.53825	9.938742	10	9		A
Silbersee (06)	51.49337	9.741578	17	15		L
Bisperode (07)	52.06686	9.537833	20	9		L
Lutterhausen (08)	51.65838	9.836403	21	21	4	L
Dobbelstein (09)	52.16024	9.166994	3	3		L
Oedingen (10)	50.61358	7.1685	20	17		A
Thueste (11)*	52.02186	9.653794	29	26	6	L
Doberg (12)*	51.99473	9.688622	3	2		A
Velbert (13)	51.49482	7.043775	11	7		A
Stolberg (14)	50.77247	6.29052	28	23	6	L
Kleinberndten (15)	51.39015	10.65258	21	19		A
Hainrode (16)	51.41864	10.65361	14	11		A
Friedrichslohra (17)	51.40187	10.62209	7	7		A
Schierschwende (18)	51.15678	10.28517	26	25		A
Deuna (19)	51.34647	10.49811	20	16		A
Herzogenrath (20)	50.82914	6.10896	14	12	6	L
Vahlbruch (21)	51.92394	9.361772	32	13		L
Huenenburg (22)	52.16153	9.154561	5	3		L
Salzhemmendorf (23)*	52.06174	9.602772	31	17	2	L
Messingberg (24)*	52.21801	9.129408	22	16		L
Tuchtberg (25)*	51.95449	9.591894	35	18	4	L
Scalyn, Belgium (26)	50.4756	5.012923	8	7		A
Malinchamps, Belgium (27)	50.19868	5.326574	11	5		A
Langenberg (28)*	51.90198	10.50589	31	20	10	L
Wolfshagen (29)*	51.91185	10.32907	30	18	4	L
Bruechs (30)	50.55617	10.15642	10	3		J
Sickenberg (31)	51.28698	10.00907	35	18	8	L
Geisleden (32)	51.36578	10.19712	3	2		A
Oberstreu (33)	50.410436	10.251206	8	0	7	J
Vinsebeck (34)	51.8387	9.0253	9	7		L
Bad Driburg (35)	51.7518	8.993	5	3		A
Bramburg (36)	51.60429	9.738438	31	19	12	L
Boneburg (37)	51.662	9.0289	8	8	2	L
Aachen (38)*	50.71057	6.197137	6	6		L

Sampling localities are numbered correspondingly in all figures, maps and manuscript text, and LIFE-BOVAR project sites are marked with an asterisk (*). Life stage information (LS) of samples per locality is shown in the final column.

*L*= larvae, *A* = adult, *J* = juvenile.

### Microsatellite loci genotyping

Individuals were genotyped for nine polymorphic microsatellite loci developed for *A. obstetricans* by Maia-Carvalho et al. (2014) (loci *Aobst_02, Aobst_06, Aobst_08, Aobst_09*) and Tobler et al. (2013) (loci *Alyobs19, Alyobs20, Alyobs23, Alyobs24, Alyobs25*) in multiplex PCR reactions (following the recommendations from the aforementioned publications) and sequenced on an ABI3130xl Genetic Analyser (Applied Biosystems). Alleles were scored in GeneMarker v3.0.1 (Softgenetics). We initially tested a larger panel of microsatellites (including ten additional loci from Maia-Carvalho et al. 2014 and seven additional loci from Tobler et al. 2013), but these were discarded due to failed amplification or because they were monomorphic. Individuals (*n* = 185) with failed amplification of more than 30% loci were discarded from further analysis, resulting in 467 genotyped individuals across 38 localities in our final dataset. Microsatellite loci were checked for deviation from Hardy-Weinberg-Equilibrium with Arlequin v.3.5.2.2 (Excoffier and Lischer 2010) (exact test, 100,000 steps Markov chain, 10,000 dememorization steps). In Micro-Checker (Van Oosterhout et al. 2004), the dataset was controlled for null alleles, scoring errors and large allele dropouts.

### Genomic library preparation and data processing

We selected a subset of 80 *A. obstetricans* extracted DNA samples from the microsatellite loci analysis representing 14 unique geographic localities across Germany to prepare genomic libraries (see Table 1). Our criteria for inclusion in the genomic libraries was to accurately represent the geographic sampling, genetic differentiation (i.e. population cluster representation based on microsatellite analysis), as well as putative founder populations. Genomic DNA was quantified using a Qubit fluorometer (Invitrogen), and equalised to a working concentration of 20 ng/ul. Genomic DNA was then digested with restriction endonucleases *SbfI* (rare cutter) and *MseI* (frequent cutter) and processed into ddRAD-seq libraries similar to the method of Truong et al. (2012). DNA fragments 300 bp to 800 bp were excised and purified using the MinElute Gel Extraction Kit (Qiagen). The final library was sent to the University of Oregon GC3F facility in Eugene (Oregon, USA) for 1×118 bp sequencing using an SP100 chip on the NovaSeq 6000. Full details of the protocol are detailed in Supplementary Material text S1. We used Stacks v.2.62 (Rochette et al. 2019) to process ddRAD-seq data and produce SNP datasets, using the *process_radtags* module to demultiplex individuals based on their individual barcodes, retaining a total of 362 million reads (mean per sample = 4.52 million, range = 62,000–8.6 million). To improve homology across samples when calling SNPs, we removed individual samples from our dataset with fewer than 2.5 million reads per sample when using Stacks, thus retaining 70 of the original 80 library samples. Following best practices (Paris et al. 2017) for Stacks we first explored our dataset using a subset of individuals (*n* = 8) from across our geographic sampling to optimise parameter settings before further analyses (see Supplementary material Text S1 for details, and Fig. S1 for a summary of data exploration). We then ran *denovo_map.pl* on the 70 samples dataset with our optimised parameters of *m* = 5, *M* = 4, retaining only SNPs present in 80% of all individuals processed, with a minimum minor allele frequency threshold of 5% (--min-maf 0.05, see Laurie et al. 2010; Ahrens et al. 2021). We ensured that SNP in the analysis were bi-allelic by interrogating the Stacks output summary files and then used a whitelist to generate specific output file formats as required for each analysis (population structure, genetic diversity and differentiation and estimating dispersal routes) by iteratively rerunning the *populations* module of Stacks.

### Population structure analysis

Population structure based on microsatellite data was inferred using the Bayesian algorithm implemented in Structure v.2.3.4 (Pritchard et al. 2000) with a burn-in period of 20,000 iterations followed by 50,000 iterations for *k* = 1–7 with 10 replicates for each *k*, which was enough to reach convergence. For all subsequent population structure analyses we also allowed k to range up to a maximum of 6 as we believe this to be an appropriate maximum number of clusters based on our knowledge of the species population structure in the south western parts of its range (Maia-Carvalho et al. 2018). Simulations assumed no admixture and no location prior, and all other parameters were set as default values. We tested the same dataset with 100,000 iterations and also with admixture and location priors, and our results are robust (see Fig. S2). The most probable number of distinct genetic population clusters was calculated with Structure Harvester (Earl and vonHoldt 2012) estimating Δk (Evanno et al. 2005) and the logarithm of the probability of the data (lnP(D | K)). Assignment probabilities of individuals to *k* = 1–6 were aligned across replicates in Clumpp v1.1.2 (Jakobsson and Rosenberg 2007) and then visualised using Distruct v1.1 (Rosenberg 2004). To validate the model-based population clustering inferred with Structure, a multivariate Discriminant Analysis of Principal Components (DAPC, Jombart et al. 2010) was applied to the dataset using the Adegenet R package (Jombart 2008). We follow the recommendations of Miller et al. (2020) for reporting DAPC results—we used the find.clusters() function in Adegenet to allow k range between 2 and 6, and chose the optimal number of clusters (*k* = 3) based on the lowest BIC (Bayesian Information Criterion) value. When determining how many PCs to retain we used the a-score, and a final number of three PCs to generate the DAPC plot based on the eigenvalues.

Using ddRAD-seq data, we investigated population structure using Admixture (Alexander et al. 2009), using k values between 2 and 6. We evaluated the likely number of population clusters represented by our data using the tenfold cross validation (CV) procedure. To complement the Admixture population structure analyses, and for comparison with microsatellite data, we additionally ran DAPC in Adegenet using the find.clusters() algorithm, again allowing *k* values to range between 2 and 6, and choosing the optimal clusters (*k* = 3) based on the lowest BIC value, using a-scores to select the number of PCs to retain, using a final number of three PCs to generate the DAPC plot.

### Genetic diversity and differentiation

Genetic diversity parameters including observed and expected heterozygosity (H_O_ and H_E_), private alleles (P_A_) and inbreeding (F_IS_) were estimated from microsatellite data using the divBasic function in the diveRsity R package (Keenan et al. 2013). Allelic richness (A_R_) was calculated with the popgenreport function from the PopGenReport R package (Adamack and Gruber 2014), also testing for departures from Hardy-Weinberg Equilibrium (using the mk.hwe=TRUE option). To maximise the number of localities we could obtain reliable genetic diversity and differentiation data for, we only calculated metrics for localities with a minimum of ten individuals (for microsatellites), and four individuals (ddRAD-seq). Localities with fewer than 4 individuals are marked with an asterisk in Table 2. We generated the same metrics based on ddRAD-seq data (H_O_ and H_E_, F_IS_), based on an 80% complete data matrix using the *--fstats* option in Stacks populations for all sites (i.e. fixed and variant) and for variant sites only. F_ST_ calculations were made with the diffCalc function of the diveRsity R package using the sample size corrected approach of Weir and Cockerham (1984), with an alpha significance level of 0.05 and 100 bootstrap replicates, calculated for microsatellite and ddRAD-seq datasets separately. Allelic richness (A_R_) and private alleles (P_A_) for each locality were calculated with rarefaction curves using ADZE (Szpiech et al. 2008) with a maximum standardised sample size of two to account for sampling unevenness. We visualised H_O_, H_E_, A_R_ and number of private alleles in geographic space using the tmap R package (Tennekes 2018). We used a Mantel test, in the vegan R package (Oksanen et al. 2012) to test for isolation by distance between Rousset’s F_ST_ (Rousset 1997, accounting for geography and population structure) and Euclidean distance between sampling localities.

**Table 2:** Genetic and genomic diversity of *Alytes obstetricans*.

	Microsatellites									ddRAD-seq				
Sampling locality	*N*	P_A_	A_R_	H_O_	H_E_	F_IS_	lower	upper	N	P_A_	A_R_	H_O_	H_E_	F_IS_
Luegde (01)	19		1.8	0.2	0.2	−0.14	−0.35	0.06						
Obernkirchen (02)	17	3	2.6	0.3	0.4	**0.18**	0.05	0.29	4(1)	0.145	1.34	0.40	0.23	−0.02
Helenenwiese (03)	20		1.4	0.1	0.1	0.23	−0.02	0.48	4	0.002	1.24	0.40	0.23	−0.06hris
Goettingen (05)	19		2.2	0.3	0.3	−0.1	−0.22	0.02						
Silbersee (06)	15		2	0.3	0.3	−0.06	−0.23	0.12						
Lutterhausen (08)*	21		2.1	0.2	0.3	**0.32**	0.18	0.45	2(2)	0.002	1.22	0.39	0.22	−0.05
Oedingen (10)	17	1	2.2	0.2	0.3	**0.18**	0.06	0.30						
Thueste (11)	26		1.8	0.1	0.2	0.42	0.31	0.54	6	0.002	1.27	0.41	0.25	−0.06
Stolberg (14)	23	2	2.4	0.3	0.4	0.29	0.15	0.42	6	0.019	1.46	0.42	0.25	−0.05
Kleinberndten (15)	19		1.9	0.2	0.2	0.01	−0.16	0.18						
Hainrode (16)	11		1.8	0.2	0.2	0.06	−0.23	0.42						
Schierschwende (18)	25		1.8	0.3	0.3	0.07	−0.08	0.21						
Deuna (19)	16		1.8	0.3	0.3	−0.11	−0.34	0.12						
Herzogenrath (20)	12		1.5	0.1	0.1	−0.16	−0.4	0.08	6	0.012	1.24	0.40	0.25	−0.04
Vahlbruch (21)	13		1.5	0.1	0.2	0.48	−0.08	0.83						
Salzhemmendorf (23)*	17		1.7	0.2	0.2	0.11	−0.14	0.37	2	0.006	1.47	0.44	0.23	0.01
Messingsberg (24)	16		2	0.2	0.3	**0.44**	0.28	0.57						
Tuchtberg (25)	18		2.1	0.2	0.3	0.36	0.19	0.50	4	0.002	1.23	0.39	0.23	−0.06
Langenberg (28)	20		2	0.3	0.3	−0.18	−0.34	0.02	5(5)	0.006	1.33	0.39	0.24	−0.05
Wolfshagen (29)	18		2.2	0.2	0.3	0.16	−0.02	0.36	4	0.035	1.54	0.43	0.23	−0.01
Sickenberg (31)	18	1	2	0.2	0.3	**0.3**	0.08	0.51	8	0.012	1.28	0.43	12	−0.06
Oberstreu (33)									6(1)	0.026	1.48	0.40	0.25	0.03
Bramburg (36)									12	0.002	1.31	0.40	0.26	−0.05
Boneburg (37)*									1(1)	0.004	1.11	0.40	0.20	0

Sampling localities are numbered correspondingly in all figures, maps and manuscript text.

*N* number of individuals included in the analysis (only localities with at least 10 individuals were used to estimate metrics for microsatellite data). For ddRAD-seq data, *N* represents numbers of individuals per locality retained for analyses, and numbers excluded (i.e. additional to the numbers retained, and not a subset of them) due to poor sequencing quality are in brackets. Asterisk represents localities where numbers of individuals used to calculate genetic diversity using ddRAD-seq data are <4 and thus should be interpreted with caution.

*A*_*R*_ mean allelic richness, *H*_*O*_ observed heterozygosity, *H*_*E*_ expected heterozygosity, *P*_*A*_ private alleles (numbers of unique alleles for microsatellites, rarefied allele percentages for ddRAD-seq data). *F*_*IS*_ inbreeding coefficient, and lower and upper Confidence Intervals (lower and upper).

Inbreeding is significant (bold) when the confidence intervals exclude zero. ddRAD-seq data is calculated from variant SNP sites only; for all sites (fixed and variant) results are shown in Table S2.

### Estimating dispersal routes

We used the estimated effective migration surfaces (EEMS) programme v.0.0.0.9000 (Petkova et al. 2016) to visualise likely dispersal routes between localities for the species. The method identifies areas of predicted gene flow and barriers, based on geography, where genetic similarity is higher (i.e. sign for gene flow) or lower (i.e. geographic barriers) than expected under isolation by distance model using spatial and SNP data. We re-ran Stacks populations to provide PLINK (Purcell et al. 2007) format output files (required by EEMS). We set the number of ‘demes’ (i.e. the number of cells in the modelling area) to 500 based on the size of our sampling area and the number of demes representing realisting units required to fill that habitat, and ran the SNP version of EEMS (runeems_snps). We used a MCMC length of 1,000,000 with a burn-in of 100,000, each for three replicates and verified that the MCMC chains had converged. We combined results using the EEMS R plotting (rEEMSplot) package and plotted surfaces of effective migration (m) rates.

### Detecting signatures of local adaptation

We scanned for potential signatures of local adaptation using a commonly used and flexible univariate Genotype-Environment Association analysis method, Redundancy Analysis (RDA, Oksanen et al. 2012), following best practices for the rda function (Capblanq and Forester 2021). To conduct our analyses, we selected two uncorrelated climate variables (Variance Inflation Factor <5) - maximum temperature of the warmest month (bioclim 5) and precipitation of the warmest quarter (bioclim 18) from the Worldclim2 dataset (Worldclim.org), as well as two landscape variables—Terrain Ruggedness Index (TRI) and Compound Topographic Index (CTI, analogous to topographic wetness index) from the Geomorpho90m dataset (Amatulli et al. 2020). We selected these four ecologically relevant variables to represent climatic extremes faced by *A. obstetricans* in the drier months, as well as accounting for topographic complexity and the availability of moisture to offer hiding places from challenging ecological conditions during the warmer summer months. We followed Razgour et al. (2019) to optimise our analyses, identifying putatively adaptive SNPs with a standard deviation of >2.5 from the mean RDA loadings. We then used the approach of Barratt et al. (2023) to categorise individual samples using only putatively adaptive SNPs as either ‘warm’, ’cold’, or ‘intermediate’ adapted and ‘rough’, ’moist’, or ’intermediate’ adapted in two separate analyses (one for warm-cold-intermediate, and one for rough-moist-intermediate). This was done using code available from the Life on the edge toolbox (https://cd-barratt.github.io/Life_on_the_edge.github.io/) which uses Genotype-Environment Association analyses to identify putatively adaptive SNPs, then categorise individuals based on their position in the RDA ordination space. With this approach it is possible to map the proportions of individuals in each locality that fall in defined adaptive categories using the mapPies function of the rworldmap R package (South 2011), providing an overview of the degree of local adaptation at each locality.

### Integrating results to create a decision-making framework for population management decisions

To integrate insights from all of the different results for *A. obstetricans* we developed a decision-making framework to inform the conservation management of populations, particularly to identify suitable donor and recipient populations for translocations or for stimulating gene flow and population connectivity where suitable, but see the discussion for some caveats when interpreting our results. Specifically, we aimed to incorporate knowledge, such as genetic diversity, population structure, local adaptations, and population connectivity of available populations and localities with landscape and habitat data (i.e. environmental suitability and stability). With this knowledge, we work towards increasing genetic diversity and evolutionary potential, whilst ensuring that population structure and local adaptations are not compromised, or that reintroductions are not made in environmentally unsuitable conditions.

## Results

### Microsatellite genotyping, ddRAD-seq data processing and SNP calling

A total of 467 individuals were genotyped for nine polymorphic microsatellite loci established for *A. obstetricans* by Maia-Carvalho et al. (2014) (loci *Aobst_02, Aobst_06, Aobst_08, Aobst_09)* and Tobler et al. (2013) (loci *Alyobs19, Alyobs20, Alyobs23, Alyobs24, Alyobs25*). For ddRAD-seq data, after parameter optimisation in Stacks we opted to select Stacks core parameters of *M* = 4, *m* = 5 and *r* = 80% for downstream analyses due to the trade-off between maximising polymorphism in our data and reducing potential ‘false’ loci caused by over- or under-merging loci. From our original (optimised but unfiltered) 2,854,228 variable sites across 636,892 loci, we removed poorly sequenced individuals (<2.5 million reads), sites with high levels of missing data across all individuals (>80%) and rare SNPs (with a minimum minor allele frequency of 5%). Our final filtered dataset contained 8650 bi-allelic SNPs for 70 individuals, using a single (the first) SNP from each locus to maintain assumptions of linkage disequilibrium for later analyses.

### Genetic diversity and differentiation

Throughout Results and Discussion we refer to locality names followed by their corresponding numbers 01-38 displayed in Tables 1 and 2, and all figures and supplementary material. Based on the microsatellite data, all localities demonstrated relatively homogeneous and low levels of genetic diversity (Table 2). Mean allelic richness (A_R_) ranged from 1.4 (Helenenwiese #03) to 2.6 (Obernkirchen #02), Observed heterozygosity (H_O_) ranged from 0.1 (Helenenwiese #03, Herzogenrath #20, Valbruch #21, Thueste #11) to 0.3 (Deuna #19, Langenberg #28, Obernkirchen #02, Bramburg #36, Schierschwende #18, Silbersee #06, Stolberg #14) and was similar to expected heterozygosity, indicating that most localities are within or close to Hardy-Weinberg equilibrium (our analysis in PopGenReport indicated no significant departures from HWE that were consistent across localities using our microsatellite and ddRAD-seq data). Most localities showed no private alleles with the exception of Oedingen #10 and Sickenberg #31 (*n* = 1), Stolberg #14 (*n* = 2) and Obernkirchen #02 (*n* = 3). However, minor signals of inbreeding (F_IS_) were detected in the Lutterhausen #08, Messingsberg #24, Obernkirchen #02, Oedingen #10, Sickenberg #31 localities.

Genomic diversity estimates using ddRAD-seq data, as expressed by mean allelic richness (A_R_) and observed heterozygosity (H_O_) were generally consistent with these patterns, with the lowest allelic richness in Boneburg #37. Unlike microsatellite analysis, observed heterozygosity was higher than expected though were also not detected via our PopGenReport analyses. Highest H_O_ was found in the Salzhemmendorf #23, Sickenberg #31, Stolberg #14, Thueste #11 and Wolfshagen #29 localities, with most other localities having similar H_O._ Private alleles were also generally evenly distributed across localities, with Oberstreu #33 and to a lesser extent, Obernkirchen #02 and Salzhemmendorf #23 showing a higher proportion of private alleles unique to those localities (Table 2, Supplementary Fig. S3). Patterns of inbreeding (F_IS_) using ddRAD-seq data were a great deal lower than those found by microsatellites, we believe due to individuals being sequenced at more loci in the ddRAD-seq dataset, similar to results obtained in other taxonomic groups; fish, Sunde et al. (2020) and birds, Zimmerman et al. (2020), respectively.

Pairwise genetic differentiation (F_ST_) based on microsatellite data (assessed with an alpha significance level of 0.05 using 100 bootstraps) was generally low to moderate between localities (0.06–0.590, mean = 0.350), with the exception of Herzogenrath #20, which was highly differentiated from the rest (0.482–0.783, mean = 0.627, Table S1). ddRAD-seq based pairwise population level F_ST_ showed similar patterns and was generally slightly lower than microsatellite-based F_ST_ (0.04–0.545, mean = 0.295), especially in our densely sampled regions from adjacent localities throughout Lower Saxony (the eastern parts of our sampling range) (Table S1). Signals of isolation by distance (i.e. higher pairwise F_ST_) were evident in geographically isolated localities at the periphery of our sampling (e.g. Herzogenrath #20 in the west, Obernkirchen #02 in the north, Oberstreu #33 in the south), confirmed with a significant Mantel test using microsatellite data (*r* = 0.404, *p* = 0.029), but non-significant using ddRAD-seq data (*r* = 0.111, *p* = 0.298).

### Population structure

Structure results suggested that *k* = 3 was a sufficient and realistic assumption to describe population structure based on the microsatellite dataset; full plots of population structure iterations between 2 and 6 are presented in the Supplementary Information (Fig. S4A). A cluster formed to the west (magenta), spanning occurrences in Belgium and North Rhine-Westphalia (localities #10, 13, 14, 20, 26, 27, 38), as well as Obernkirchen #02, which is the northernmost locality examined. In the centre of this study area, which extends across Lower Saxony, Thuringia and the east of North Rhine-Westphalia, a cluster (green), represented by localities #01, 07, 08, 11, 12, 21, 22, 23, 25, 34, 35, 37, was divergent from the remaining eastern population cluster (yellow), though with some degree of admixture between localities.

**Fig. 2:**
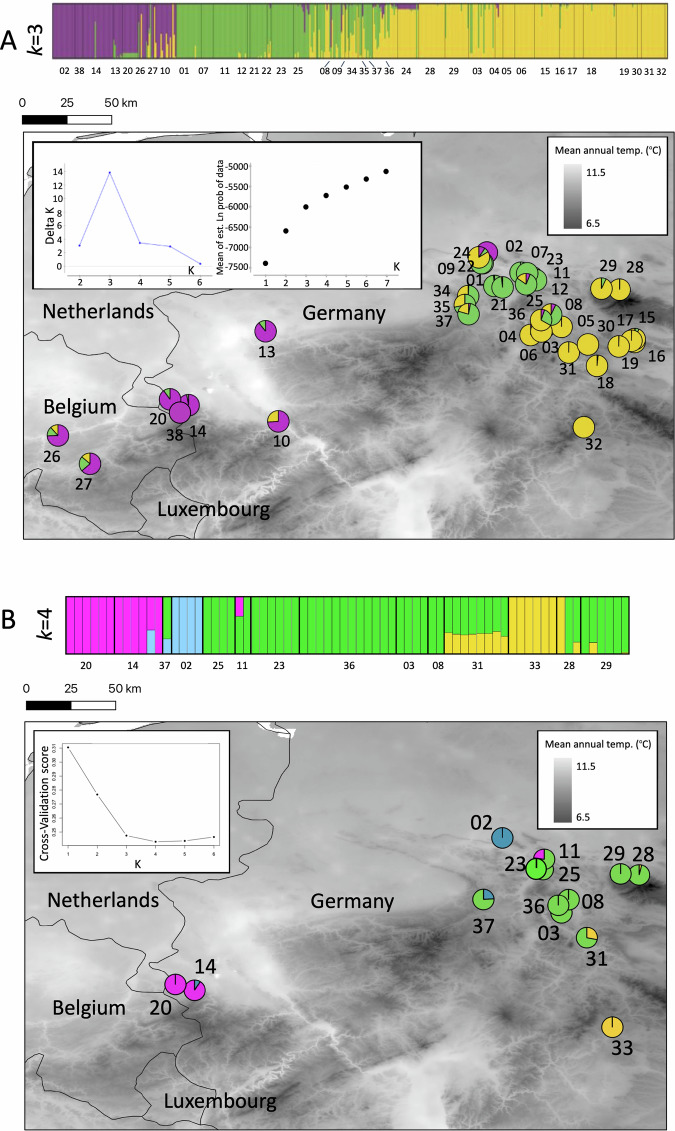
Population structure of Alytes obstetricans. Population structure of *Alytes obstetricans* throughout our sampling region based on **A** Microsatellites and **B** ddRAD-seq data. Population clusters are coloured based on their co-ancestry matrices (upper panels) and represented geographically (lower panels, sampling locality numbers follow Table 1). For Microsatellites the results from Structure were averaged over the ten iterations in Clumpp and visualised in Distruct.

ddRAD-seq based Admixture results showed that between 3–6 population clusters satisfactorily describe the data (see Fig. 2B), with lower CV scores for these iterations of k and with a local maximum for *k* = 6. The main plot shows *k* = 4 as this was the lowest CV score. Full plots of population structure iterations between 2 and 6 can be found in the Supplementary Information (Fig. S4B). Western localities (Herzogenrath #20 and Stolberg #14) formed their own non-admixed (magenta) population cluster, in addition to a non-admixed cluster for the northern locality of Obernkirchen #2 (blue), similar to the microsatellite loci analyses but with clearer differentiation between population clusters (i.e. less shared co-ancestry). Contrary to the microsatellite loci results, ddRAD-seq data suggested that geographically proximal localities towards the centre of our sampling area (#11, 23, 25, 28, 29, 08, 36, 03, 37, 31) formed a single large population cluster (green), with the latter demonstrating some shared admixture with individuals from Sickenberg #31. Similarly, some individuals from our southernmost sampled locality (Oberstreu #33 yellow cluster), and Wolfshagen #29 and Langenberg #28 also shared ancestry with Sickenberg #31. Together, the ddRAD-seq data tended to show slightly stronger population structuring rather than the continuum of differentiation shown by the microsatellite loci, though gene flow appeared to be fairly unimpeded in most cases. DAPC analyses generated largely congruent results with the Structure and Admixture analyses, with microsatellites showing a continuum of divergence and ddRAD-seq data showing higher population structure (Fig. S5).

### Estimating dispersal routes

**Fig. 3:**
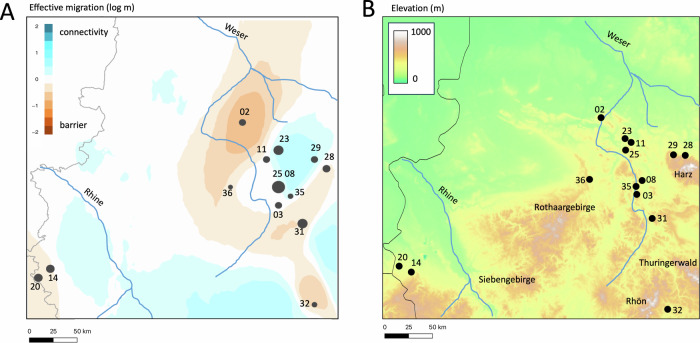
Estimated effective migration and geographic barriers. **A** Estimated effective migration surfaces based on ddRAD-seq data. Sampling localities represented by black dots, sample sizes per locality proportional to size of dots. Note that black dots of deme locations in EEMS outputs are plotted on the map based on the number of demes assigned in the analysis, so several localities are collapsed into a single central dot and thus do not exactly match the real geographic locations. Blue areas represent genetic connectivity, orange areas represent genetic barriers. **B** Elevational profile of the study region showing main mountain ranges. The approximate courses of the major rivers in the region are overlaid as blue lines in both panels (Weser and Rhine). Population numbers follow Table 1.

EEMs analysis supported the population structure results, with no obvious strong barriers found that may geographically structure the sampled *A. obstetricans* localities (Fig. 3A). The similarity in the central localities was reflected by an area of higher gene flow, which is in some cases disrupted by potential landscape barriers (Fig. 3B). This is also reflected by the relatively higher F_ST_ values between these and the remaining sampled localities (e.g. Obernkirchen #02, Herzogenrath #20, Oberstreu #33 and Boneburg #37, see Supplementary Material Table S1). Moreover, the differentiation observed for Obernkirchen #02, which is thought to be founded by introduced individuals from a locality in Northern France (Buschmann et al. 2006) and its increased differentiation from other localities (population structure, higher F_ST_ values) may further contribute to the observed barrier that separates Herzogenrath #20, Stolberg #14 from the remaining central and eastern localities across our sampling regime.

### Signatures of local adaptation

All four of our environmental predictors remained after testing for collinearity using Variance Inflation factors <5. Of the 8650 SNPs tested for signals of local adaptation, a total of 177 candidate SNPs were detected with a standard deviation of over 2.5 from the mean loadings using RDA (43 candidates potentially linked to bioclim_5, 16 loci to bioclim_18, 18 loci to CTI, and 100 loci to TRI), suggesting a stronger effect of temperature and terrain ruggedness than that of rainfall and topographic wetness. We plotted all SNPs in an ordination plot and colour coded them based on their environmental associations with our four environmental predictors, accounting for the fact that the SNPs may be both positively or negatively associated with the relevant predictor (Fig. S6).

**Fig. 4:**
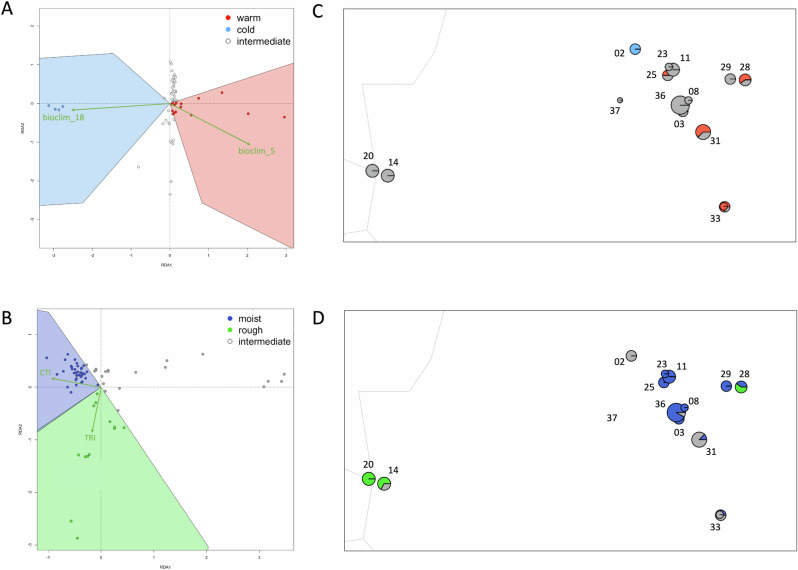
Local adaptation results based on RDA analysis (based on putatively adaptive SNPs that are >2.5 st. dev. from the mean loadings). Sampling locality numbers follow Table 1. **A** Warm/cold/intermediate individual categorisation based on RDA positions relative to environmental predictors (bioclim_5, bioclim_18). **B** Moist/rough/intermediate individual categorisation based on RDA positions relative to topographic predictors (Compound Topographic Index, Terrain Ruggedness Index). **C** Spatial mapping of local adaptations and D to environmental predictors (bioclim 5, bioclim 18) and topograpic predictors (Compound Topographic indexbased on ddRAD-seq data). **D** Spatial mapping of local adaptations to topographic predictors (Compound Topographic Index, Topographic Roughness Index) based on ddRAD-seq data. Spatial variation of each predictor across the study landscape can be found in Fig. S6.

Categorising individuals based on their position in the RDA ordination space using only putatively adaptive regions of the genome (i.e. the RDA candidate SNPs), showed that in general, several localities showed signals of local adaptation, but these were not overwhelmingly dominant. A handful of individuals were adapted to one of the warm or cold temperature axes, and the majority of localities were adapted to moist conditions or rough topography (Fig. 4). For the climate data (bioclim_5 and bioclim_18), we parsed 15 individuals that were ‘warm’ adapted, 4 that were ‘cold’ adapted, and 51 ‘intermediate’ that were not adapted to either set of conditions (Fig. 4A, C). Analysing Genotype-Environment Associations with topographic data (TRI and CTI), 37 were ‘rough’ adapted, 13 were ‘moist’ adapted, and 20 individuals were intermediate (Fig. 4B, D). Mapping these individuals proportionally for each locality in geographic space showed that most localities are intermediate, some are partially warm adapted (Tuchtberg #25, Sickenberg #31, Langenberg #28, Oberstreu #33), and Obernkirchen #02 is cold adapted. In terms of topographic predictors, the eastern and westernmost localities (Herzogenrath #14, Stolberg #20, Langenberg #28) are rough adapted, and the majority of the central localities (Bramburg #36, Lutterhausen #08, Tuchtberg #25, Thueste #11, Salzhemmendorf #23, Wolfshagen #29) have over 75% of their sampled individuals adapted to moist conditions, with weaker signals of moist-adapted individuals (<50% of their sampled individuals) in the Oberstreu #33, Sickenberg #31 and Langenberg #28 localities. To visualise the putative adaptive landscapes we detected for *A. obstetricans*, a summary of the spatial variation of each of the four predictors used in our GEA analyses in relation to ddRAD-seq population sampling can be found in Fig. S7.

## Discussion

**Fig. 5:**
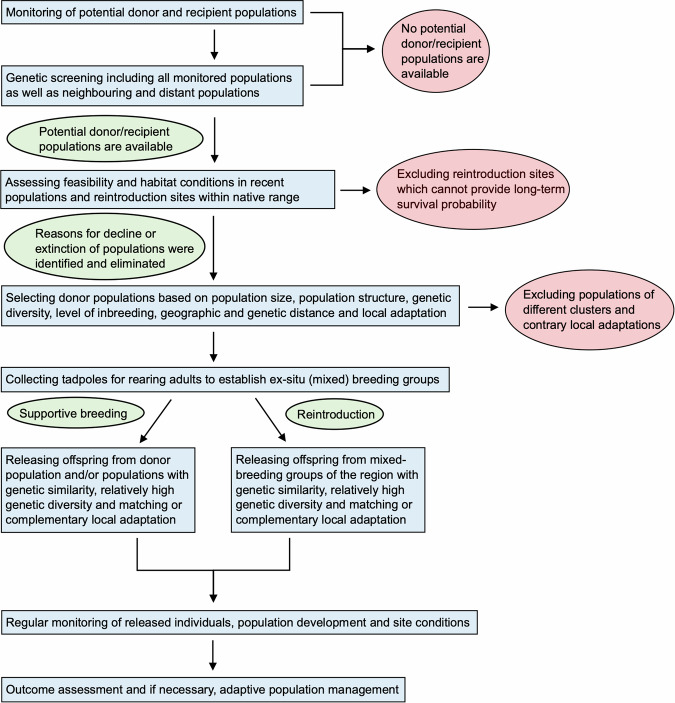
Workflow diagram for decision-making based on IUCN guidelines and information on population status and structure, genetic diversity, local adaptation and connectivity of *Alytes obstetricans* localities and populations.

Focusing on *Alytes obstetricans*, the common midwife toad, at its range limits in Europe, we developed an integrative decision-making framework based on genetic and genomic data to maximise evolutionary potential across localities. Using a combination of microsatellite and ddRAD-seq markers we demonstrated that *A. obstetricans* localities throughout Germany and eastern Belgium are weakly genetically differentiated, with generally homogenous patterns of genetic diversity (though with some notably higher genetically diverse localities), minor signals of local adaptation and in general no major strong barriers to gene flow, though some mountains and rivers likely contribute to the maintenance of current population structure. Accounting for these combined insights, we discuss the results and interpretation of our analyses in terms of conservation management applications. In doing so, we outline a decision-making framework (Fig. 5) to assist in identifying localities of conservation concern (i.e. those with low diversity that are also geographically and genetically isolated), increasing genetic diversity and evolutionary potential in general, as well as earmarking suitable donor and recipient populations and localities for evolutionary rescue. However, we acknowledge that sample sizes are low for our ddRAD-seq data and that in future more comprehensive sampling per population would explore the observed patterns better and enable better information for conservation management efforts.

### Low but significant genetic diversity and local adaptations may help to increase evolutionary potential

Negative environmental impacts on *A. obstetricans* at its range limits across Germany may be attributed to habitat loss and modification, in particular changes in availability of suitable terrestrial and aquatic habitats (Gasc 1997), which has led to the local extirpation of many populations and the species endangered status on the IUCN red list for Germany (Scheidt 2020). Accordingly, genetic differentiation and genetic diversity is relatively low in this part of the species range when compared with the remainder of its range (e.g. in southern and western Europe through France, Spain and Portugal, Goncalves et al. (2015)), Dufresnes and Martínez-Solano (2020), Lucati et al. (2022), Ambu et al. (2023). We speculate that the low genetic diversity in these peripheral range localities of *A. obstetricans* seemingly represent signals of gradual and accumulated genetic erosion due to a combination of range expansions from glacial refugia, incorporating founder effects, population bottlenecks and lack of intermixing with populations with high genetic diversity (e.g. Maia-Carvalho et al. 2018), a common pattern evident in other European amphibian species (Dufresnes and Perrin 2015; Bernabò et al. 2022). Despite the relative similarity in genetic diversities across localities in our study area compared with the species across its range, we nevertheless detect very slightly heterogeneity between sampled localities, with higher observed heterozygosity estimates for Salzhemmendorf #23, Sickenberg #31, Stolberg #14, Thueste #11 and Wolfshagen #29 (based on ddRAD-seq data) and Deuna #19, Langenberg #28, Obernkirchen #02, Bramburg #36, Schierschwende #18, Silbersee #06 and Stolberg #14 (based on microsatellite data). This information can help to guide conservation management actions to increase population numbers, viability and evolutionary potential, for example to avoid the possible dilution of recipient gene pools with the introduction of genetically impoverished individuals.

### Implications of local adaptation and population history

In addition to considering neutral genetic diversity alone, a major consideration for managing evolutionary potential is the degree of local adaptation across populations and how this can be leveraged to increase the survival probabilities of individuals and populations (Harrisson et al. 2014; Hoelzel et al. 2019; Milot et al. 2020). Generally speaking, if population structure and differentiation is natural (e.g. as a result of demographic history and landscape barriers), and local adaptation is not strong, mixed breeding groups could be formed using multiple localities within the same genetic clusters, where shared similar past responses to environmental changes between localities are potentially more likely to lead to the formation of viable and resilient populations. Alternatively, if population structure and differentiation are driven mainly by recent bottlenecks or reductions in local population sizes due to human disturbance and fragmentation, wider mixing (across different population clusters) may help to restore genetic diversity that has been more recently lost.

Our combined results for *A. obstetricans* suggested the former scenario, that the majority of localities are not strongly locally-adapted, and our interpretation of the patterns of genetic differentiation is that they are mostly natural due to geographical distance and barriers (mountains and rivers) between localities. Thus, for the practical conservation of *A. obstetricans* at its eastern and northern range limits, we advocate the formation of mixed breeding groups from localities belonging to the same broadly inferred genetic clusters. In this way, via translocations or efforts to promote natural gene flow by creating migration corridors, genetic diversity can be increased in the context of supportive breeding and reintroductions and thus also the adaptability of populations, e.g. with regard to climatic changes or diseases. However, a caveat is that our genomic data is limited to a handful of individuals per locality, with only a small proportion of the genome (i.e. ddRAD-seq as opposed to high coverage WGS data), and based on four ecologically informed environmental predictors, so our local adaptation and genetic diversity results should be seen as preliminary rather than conclusive. Furthermore, we made attempts to quantify effective population size, both contemporary (Do et al. 2013) and historical (Barbato et al. 2015) but unfortunately the estimates and their confidence intervals were consistently unreliable for management purposes.

With this in mind, population management decisions should ideally take into account other local adaptations to environmental and ecological conditions beyond those that are tested here. For example, *A. obstetricans* has one of the highest prevalences of the pathogenic fungus *Batrachochytridium dendrobatidis* (*Bd*, Ohst et al. 2013) within German amphibian species, infecting up to 20% of sampled populations, but this has not been attributed as a cause of population declines. There remains an intriguing possibility that *Bd* resistant and resilient genotypes may exist in certain populations and localities, which would also be important to account for when considering making management decisions (including translocations) to increase evolutionary potential and resistance to pathogens. Based on the genetic findings of this study, we have established two mixed breeding groups from reared tadpoles within the LIFE BOVAR project: Salzhemmendorf #23 and Thueste #11 as well as Wolfshagen #29 and Langenberg #28. Salzhemmendorf and Thueste occur in the region Weser-Leinebergland, belong to the same population cluster and have a low pairwise genetic distance. Wolfshagen and Langenberg from the Harz region, show genetic similarity, also a low genetic distance and shared ancestry. Within both mixed breeding groups, the genetic diversity (H_O_, A_R_, P_A_) and inbreeding coefficient (F_IS_) were broadly similar but differed slightly between populations. Concerning local adaptation, Salzhemmendorf #23 and Thueste #11 were ‘intermediate’ with regard to climate data but they showed some adaptation signals to moist conditions. Wolfshagen #29 and Langenberg #28 also have moist adapted individuals and Langenberg #28 showed signals of adaptation to rough topography and warmer conditions. Consequently, mixed breeding groups of these localities with genetic similarity as well as matching and complementary local adaptations but differing in genetic diversity and levels of inbreeding could increase the viability and evolutionary potential of the recipient populations by supportive breeding as well as that of reintroduced populations.

However, the selection of donor populations and the establishment of mixed breeding groups must be carefully considered. As a case in point, Tuchtberg #25 and Obernkirchen #02 localities, with their similar quarry habitats and patterns of genetic diversity, demonstrated that they belong to different population clusters. Furthermore, divergent signals of local adaptations to climatic conditions were evident, with half of the sampled individuals in Tuchtberg being categorised as warm adapted and all sampled individuals in Obernkirchen categorised as cold adapted, indicating that the mixing of these localities would not be advisable from a conservation perspective based on available evidence. Highlighting the importance of knowledge about population history and integrating this in the decision-making process for management actions, we also have reason to believe that the individuals at Obernkirchen were founded by an introduction from northern France several years ago by an amateur herpetologist (Buschmann et al. 2006), which would explain much of the observed differentiation of this locality from its nearby conspecifics. However, this potentially non-autochthonous locality may be seen as viable and highly adaptable, and appaently, stable. From an evolutionary perspective, this locality highlights the high evolutionary potential and adaptability of *A. obstetricans*. Despite potentially stemming from an introduction, it has no lesser value in terms of conservation than individuals in localities that are presumed to be autochthonous, though as it forms a separate genetic cluster from our remaining localities we advise against actively promoting gene flow with other populations.

### Integrated approaches to maximise the success of conservation management efforts

To improve the population numbers and viability of species at their range limits, efforts must be made to improve habitat availability and population connectivity whilst maintaining a sufficient and diverse mix of neutral and adaptive genetic diversity of their individuals. For *A. obstetricans* this necessitates (re)building a gene pool that has been impoverished by a combination of both natural and anthropogenic processes, whilst maintaining relatively stable metapopulation structure and dynamics and avoiding inbreeding depression in local populations and localities. These are challenging problems to address, but by integrating ecological, environmental and molecular information with efforts to restore habitats and knowledge of species ecology and population origins we may work towards increasing the genetic diversity and evolutionary potential of threatened and non-threatened species to safeguard their future viability with a quantitative and evidence-based approach. To this end, we developed a general decision-making framework to help with this process, which we applied to *A. obstetricans*. Our framework, relying on empirical ecological, environmental and molecular data is applicable to other amphibian species and other taxonomic groups when available data is on hand to support selected conservation management actions. Though we were able to inform conservation management actions using our *A. obstetricans* data we would suggest that caution is taken with our results due to being based on relatively small sample numbers (in the case of our ddRAD-seq data in particular). Further sampling across the range edges of *A. obstetricans* using larger numbers of individuals per locality and ideally using WGS approaches would help to evaluate the robustness of the analyses presented here.

In the future, modelling approaches would benefit from integrating aspects of our framework with existing tools to simulate the effects of different management strategies before making decisions on final management actions. Outcomes of these simulations may help to inform on habitat suitability given future environmental predictions using species distribution models, landscape genetics to model connectivity between localities, populations and habitat patches, and spatially explicit eco-evolutionary simulations of genetic diversity, effective population size and inbreeding (e.g. Landguth et al. 2017; Haller and Messer 2019).

## Supplementary information

Supplementary material: A decision-making framework to maximise the evolutionary potential of populations - Genetic and genomic insights from the common midwife toad (Alytes obstetricans) at its range

## Data Availability

Code and data (including microsatellite and SNP genotypes with all associated metadata) are accessible in a DRYAD repository containing input files for all analyses and code in an accompanying ZENODO repository so all aspects of this work can be reproduced (10.5061/dryad.x69p8czt6). Demultiplexed sequence data is available at the European Nucleotide Archive project number PRJEB77624. Benefits Generated: A collaboration was developed with scientists from the countries providing genetic samples (Germany and Belgium), all collaborators are acknowledged alongside the co-authors, and the results of research have been shared with the provider communities and the broader scientific community. The research addresses a priority concern, in this case the conservation of *Alytes obstetricans*, but is applicable to other endangered species. Our group is committed to national and international scientific partnerships, as well as institutional capacity building.
