# Supplementary material for: A decision-making framework to maximise the evolutionary potential of populations - Genetic and genomic insights from the common midwife toad (Alytes obstetricans) at its range limits
Source: Heredity (Edinb). 2024 Sep 2;133(4):249–61. doi: 10.1038/s41437-024-00710-4 (PMC11436998; doi:10.1038/s41437-024-00710-4)
Supplement: Supplementary file 1 — Supplementary material: A decision-making framework to maximise the evolutionary potential of populations - Genetic and genomic insights from the common midwife toad (Alytes obstetricans) at its range [file 41437_2024_710_MOESM1_ESM.docx]

**Supplementary material: A decision-making framework to maximise the evolutionary potential of populations - Genetic and genomic insights from the common midwife toad (*Alytes obstetricans*) at its range limits**

Christopher D. Barratt^1,2,3^, Kathleen Preißler^4^, Pauline R. Jennert^4^, Falk Eckhardt^5^, Mirjam Nadjafzadeh^5^, Sebastian Steinfartz^4^

^1^ German Centre for Integrative Biodiversity Research (iDiv) Halle-Jena-Leipzig, Puschstrasse 4, 04103 Leipzig, Germany

^2^ University of Leipzig, Ritterstrasse 26, 04109, Leipzig, Germany

^3^ Naturalis Biodiversity Center, Darwinweg 2, 2333 CR Leiden, the Netherlands

^4^ University of Leipzig, Institute of Biology, Molecular Evolution and Systematics of Animals, Talstrasse 33, 04103 Leipzig, Germany

^5^ NABU (Nature and Biodiversity Conservation Union) Lower Saxony, Alleestrasse 36, 30167 Hannover, Germany

**Supplementary Material**

**Text S1.**

*Chloroform-Isoamyl alcohol DNA isolation protocol*

To extract high quality DNA from our samples, we modified the protocol from Gilbert et al. (2007) as below:

C digestion buffer

- 3 mM CaCl2
- 100 mM Tris (ph 8)
- 100 mM NaCl
- 2 % SDS (Sodium dodecyl sulphate)
- 40 mM DTT (Dithiotreitol)

Protocol

Preheat C digestion buffer to 55 °C.

- Crush sample if necessary
- Add 500 µl C digestion buffer and 100 µl proteinase K. Vortex.
- Incubate overnight at 55 °C on a shaking platform.

Cool down Centrifuge to 4 °C.

- Add 10 µl RNase A (10 mg/ml). Vortex.
- Incubate at 37 °C for 30 min.
- Add 1 Volume (550 µl) chloroform- isoamyl alcohol (24:1)
- Vortex 10-20 sec.
- Centrifuge at 14.000 rcf for 3 min at 4 °C.
- Carefully pick up upper phase with pipette and transfer in new tube.
- Repeat step 2.
- Add 1/5 Volume (100 µl) 4M LiCl. Vortex.
- Add 2/3 Volume (400 µl) isopropanol
- Store at -20 °C for at least 30 min (preferably overnight).

Cool down Centrifuge to 4 °C. Preheat thermomixer to 41 °C.

- Centrifuge 30 min at 12.000 rcf at 4 °C.
- Carefully pick up liquid phase with pipette to not destruct DNA pellet at bottom side of tube and discard.
- Add 500 µl ethanol (70%, cooled).
- Centrifuge 15 min at 12.000 rcf at 4 °C.
- Carefully pick up liquid phase with pipette to not destruct DNA pellet at bottom side of tube and discard.
- Repeat step 2.
- Dry DNA-Pellet in SpeedVac for 10 min at 30 °C.
- Add 100 µl TE buffer
- Incubate 1 h at 41 °C on shaking platform.
- Store DNA at -20 °C or -80 °C.

*ddRAD-seq library preparation*

Genomic DNA from the population was digested with restriction endonucleases *SbfI* (rare cutter) and *MseI* (frequent cutter) and processed into ddRAD-seq libraries similar to the method of Truong et al. 2012. Briefly, ~125 ng of genomic DNA was digested for 120 min at 37°C in a 20 μL reaction with 2.5 units of *SbfI* and 2.75 units of *MseI* (New England Biolabs [NEB]).

After digestion, samples were heat-inactivated for 10 min at 80°C followed by addition of 1.25 μL 1 μM “P1” *PstI/SbfI* adapter and 0.1 μL 250 μM “P2” *MseI* adapter. P1 adapters each contain a unique multiplex sequence index (barcode) which is read during the first five nucleotides of the Illumina sequence read. P1 and P2 adapters were added to each sample along with 36 units of T4 DNA Ligase (high concentration [HC], Enzymatics, Inc), 0.3125 units of *MseI*, 0.25 units of *SbfI* (NEB), RL Buffers A and B (Keygene) and 10 mM ATP in a final reaction volume of 25 μL, which was then incubated at 37°C for 180 minutes. Samples were diluted 1:10 in water and 2.5 μL of this product was used in a PCR amplification with 10x PCR Buffer 1 (Applied Biosystems), 0.1 μL 20 nM dNTP mix, 0.3 μL of 50 ng/μL MseI primer, 0.05 μL of PstI primer (compatible with SbfI-ligated ends), and 0.2 units of AmpliTaq DNA Polymerase (Applied Biosystems). The PCR program was as follows:

2 min 72°C

13 cycles of:

- 30 sec 94°C
- 2 min 67°C, decreasing -0.7°C per cycle
- 2 min 72°C

37 cycles of:

- 30 sec 94°C
- 2 min 58°C
- 2 min 72°C

Hold 4°C

The amplified product from each sample was pooled and mixed thoroughly. 125 μL of the pooled product was purified with a MinElute Enzymatic Reaction Cleanup Kit (Qiagen), eluted in 15 μL, and run on a 1.5% agarose gel. DNA 300 bp to 800 bp was excised and purified using the MinElute Gel Extraction Kit (Qiagen).The final library was sent to the University of Oregon GC3F facility in Eugene (Oregon, USA) for 1x118 bp sequencing using an SP100 chip on the NovaSeq 6000.

*ddRAD-seq Stacks parameter optimization*

We ran *denovo_map.pl*, iterating across a range of values of m (minimum depth of coverage required to create a stack, set between 3-10), M (number of mismatches allowed between stacks within individuals, set between 1-9) and r (minimum percentage of individuals in a locality required to process a locus for that locality, set at maximum 60, 40 and 20% missing data permitted). From each of the 72 *denovo_map.pl* runs we extracted the total number of assembled sites, total number of polymorphic sites and percentage (%) of polymorphic loci as well as the total number of SNPs and used this to assess the optimal parameter combination to run the complete dataset.

Across our test runs of denovo_map.pl to optimize parameter settings, total numbers of assembled sites, number of polymorphic sites, % polymorphic loci and total SNPs varied substantially, predictably with higher amounts of sites, loci and SNPs as parameters were relaxed (i.e. higher M values - more mismatches allowed between stacks within individuals, lower m values – greater numbers of lower coverage loci, lower r values – more missing data permitted). Our aim was to maximise informative loci and SNPs rather than focusing on sheer numbers of possible confounded data, thus we focused on the r80 datasets (i.e. max 20% missing data) as recommended by Paris et al. (2016). Within the higher quality r80 datasets, assembled sites, polymorphic sites and numbers of SNPs were actually higher with lower values of M (i.e. fewer mismatches permitted), which may be due to paralogous loci being over filtered, especially given the estimated large genome size of *A. obstetricans* (between 8 and 9.78 Gb, see <https://www.genomesize.com/>).


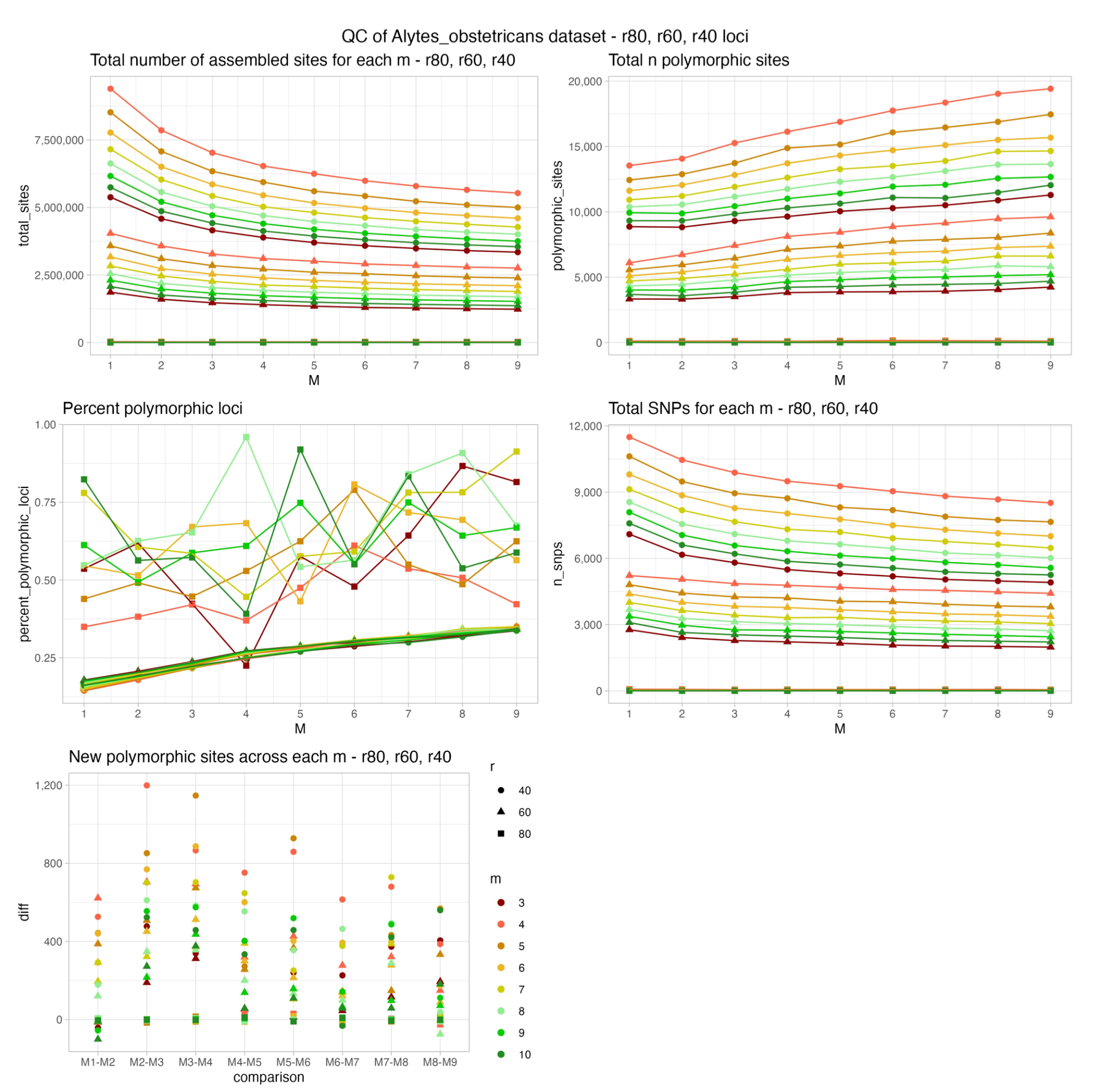


**Fig. S1.** Parameter optimization in Stacks. First four panels represent total number of assembled sites, number of polymorphic sites, percent polymorphic loci and total SNPs for each value of m in Stacks. Final panel shows the number of new polymorphic sites attained when increasing m across different parameter combinations. All plots show maximum % missing data permitted (r80 = squares, max. 20%, r60 = triangles, max. 40%, r40 = circles, max. 60%), and for each replicate of m (coverage of Stacks loci between 3 and 10, represented by colours) and M (1-9, x axis).


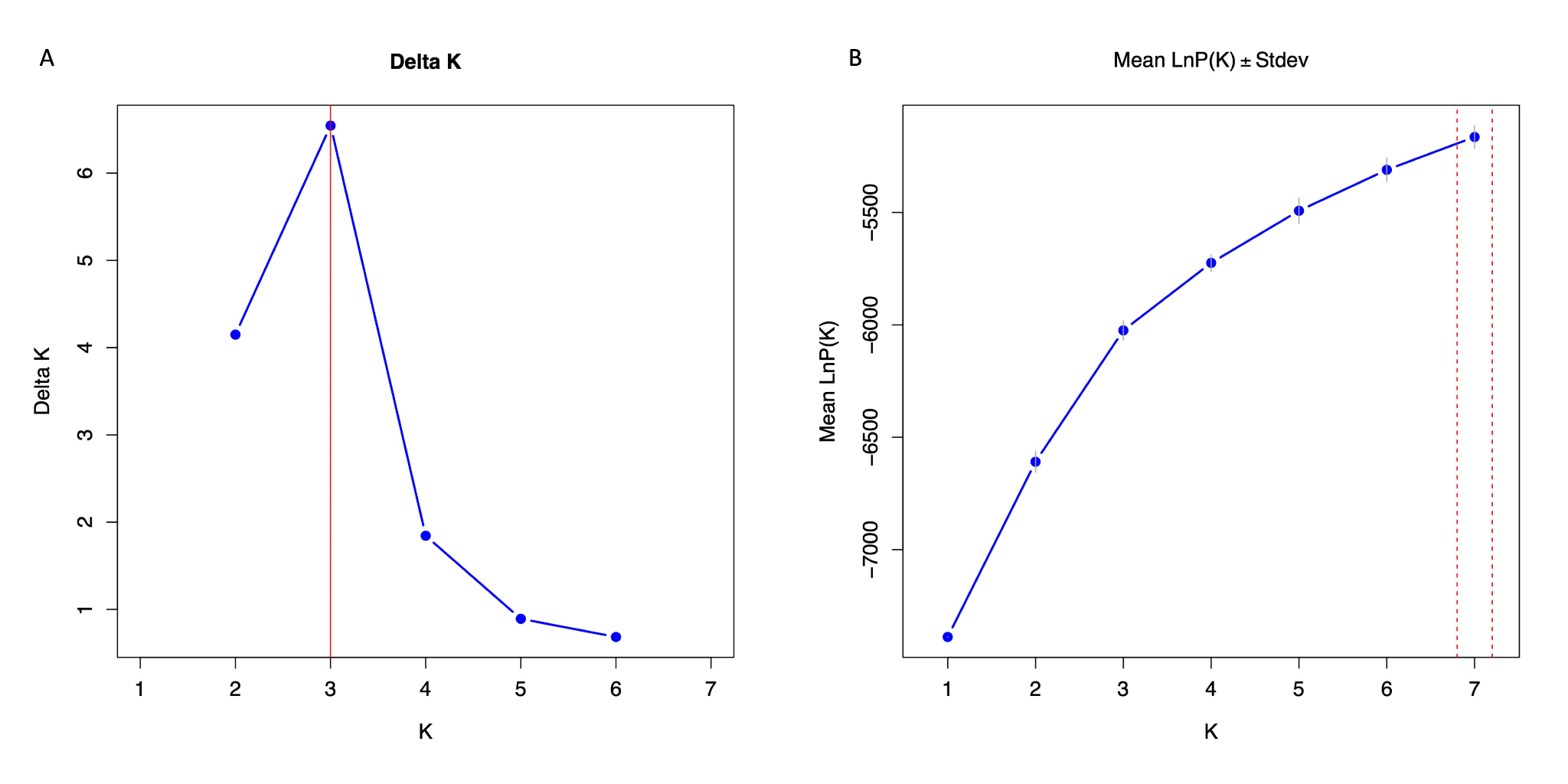


**Fig. S2.** Population assignment for Structure analysis of microsatellite data, using 100,000 iterations, including admixture and location priors.


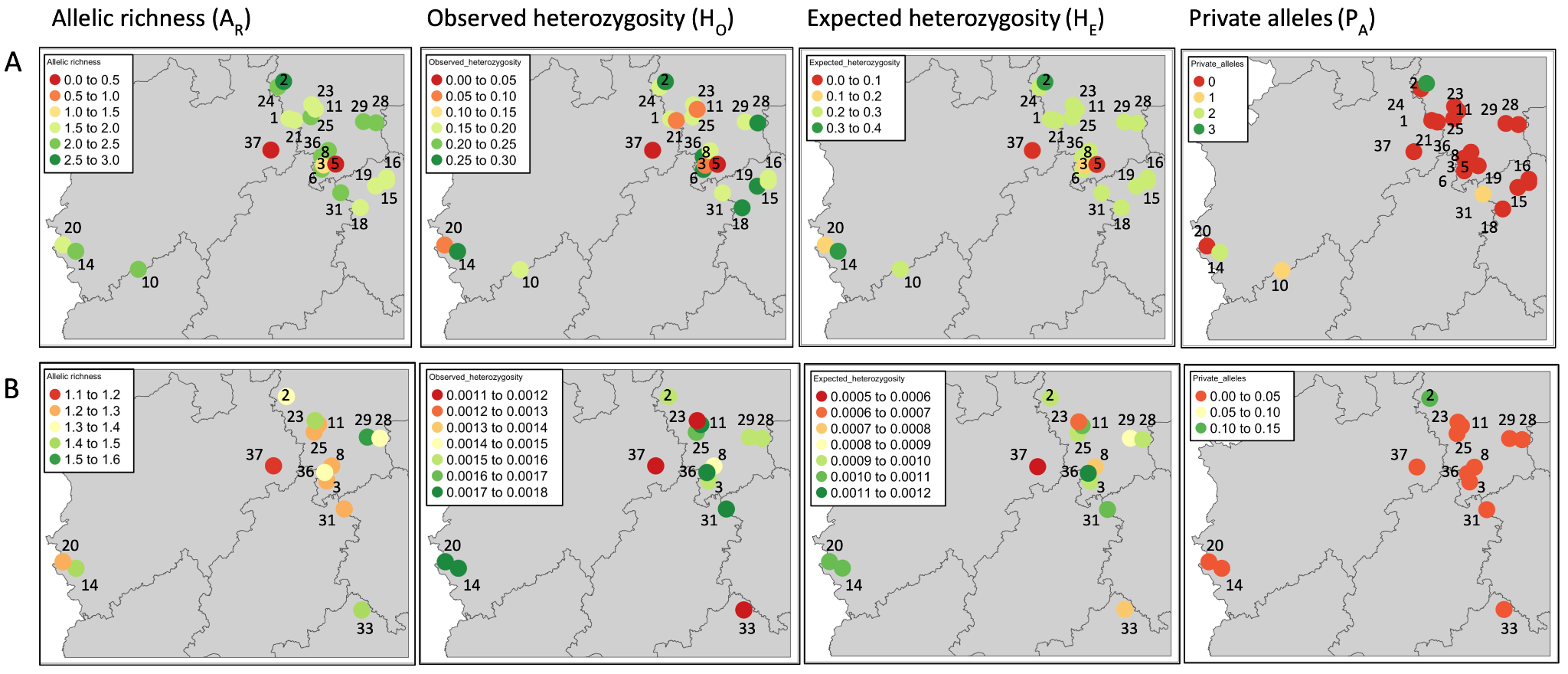


**Fig. S3.** Maps of Allelic richness, observed and expected heterozygosity, and private alleles, using A) microsatellite and B) ddRAD-seq data. Localities are numbered matching Table 1, colours represent variation in metrics across localities (legends inset for each panel).


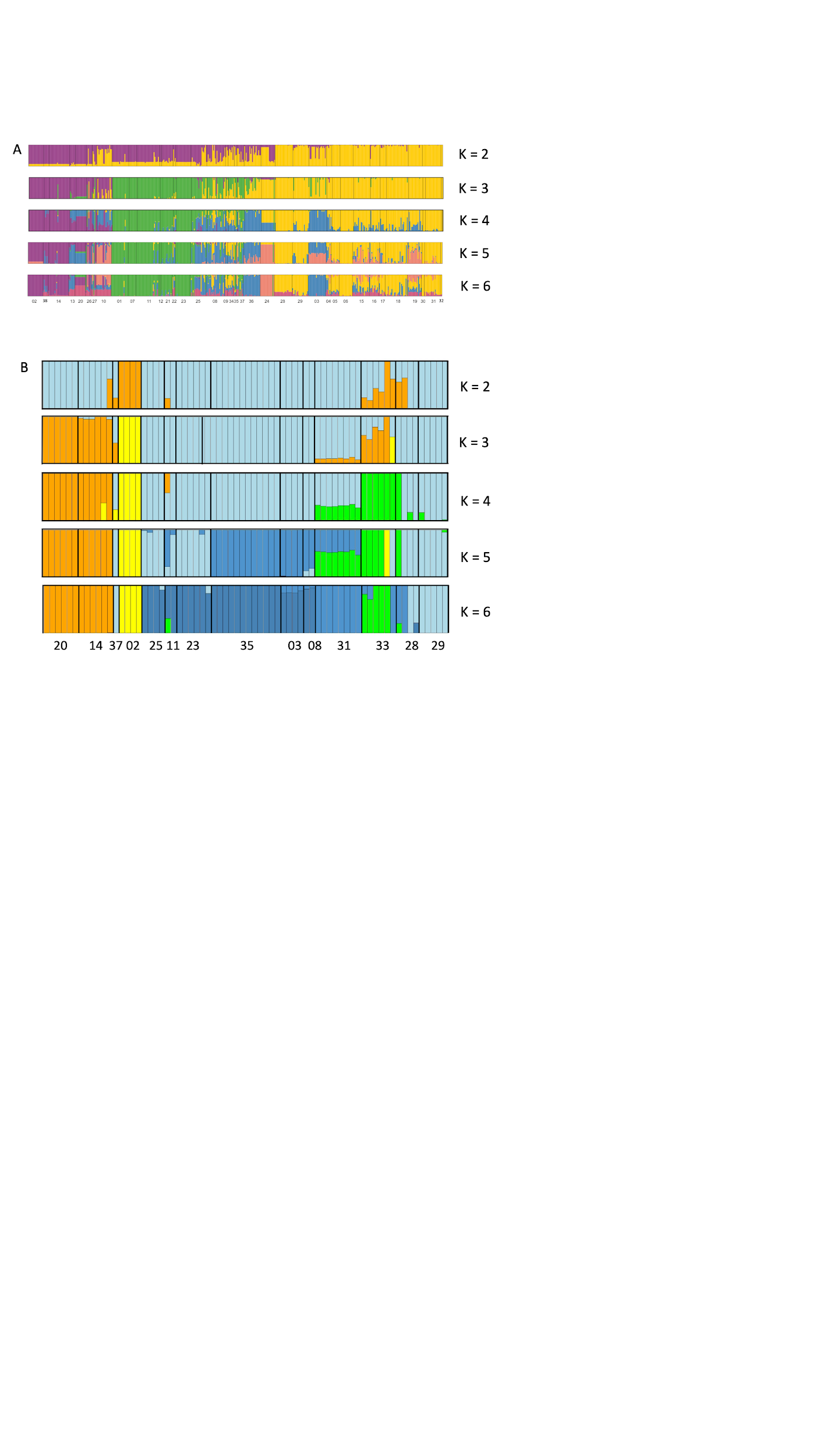


**Fig. S4.** Population assignment for k between 1 and 6 using A) microsatellites, B) ddRAD-seq data. Numbers of localities match Table 1 in the main manuscript.


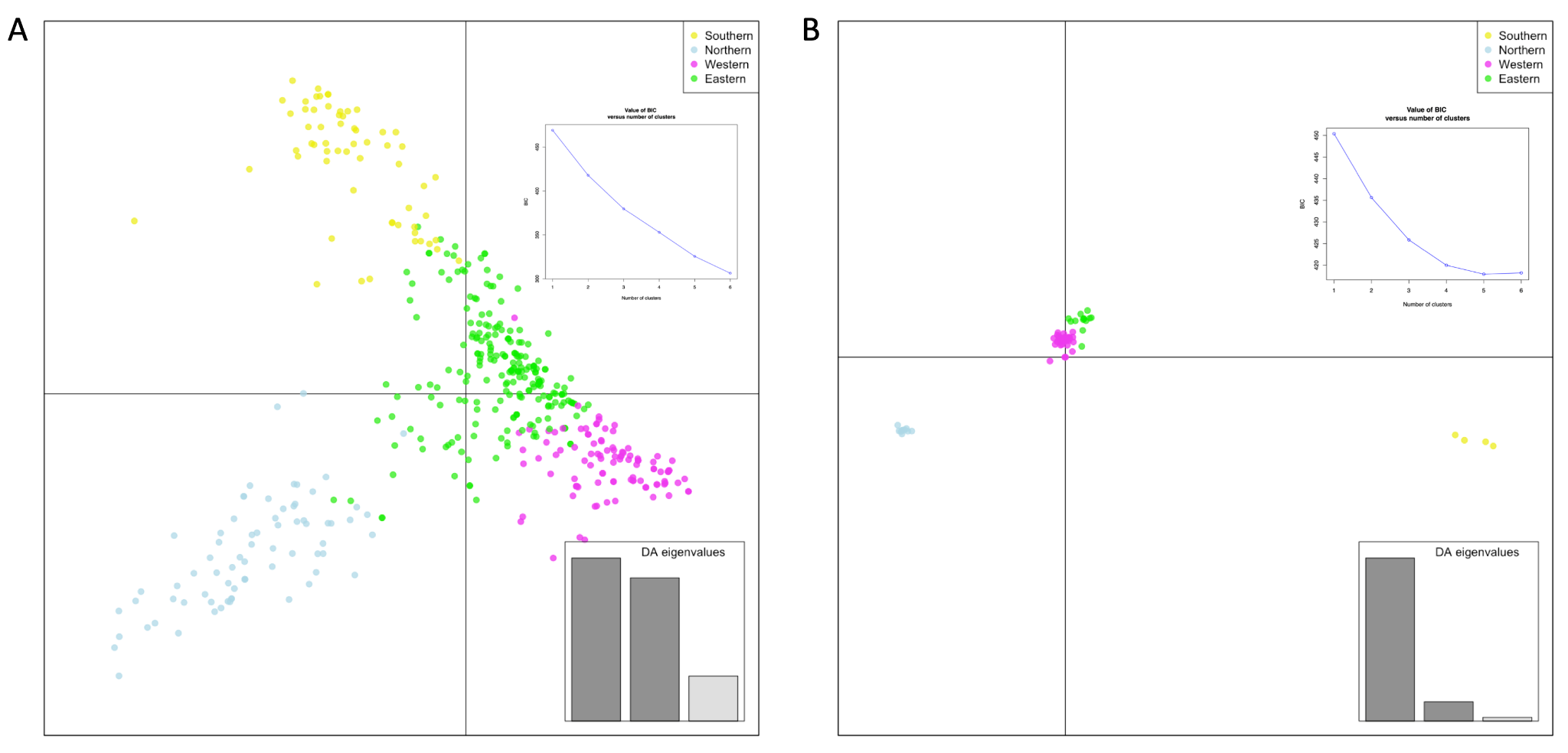


**Fig. S5.** Discriminant Analysis of Principal Components based on A) microsatellites (467 individuals) and B) ddRAD-seq data (70 individuals). Population clusters coloured to match Fig. 3 in main text, Inset panels show Bayesian Information Criterion (BIC) values for number of population clusters (k=1-6) and eigenvalues for each discriminant function retained in the DAPC.


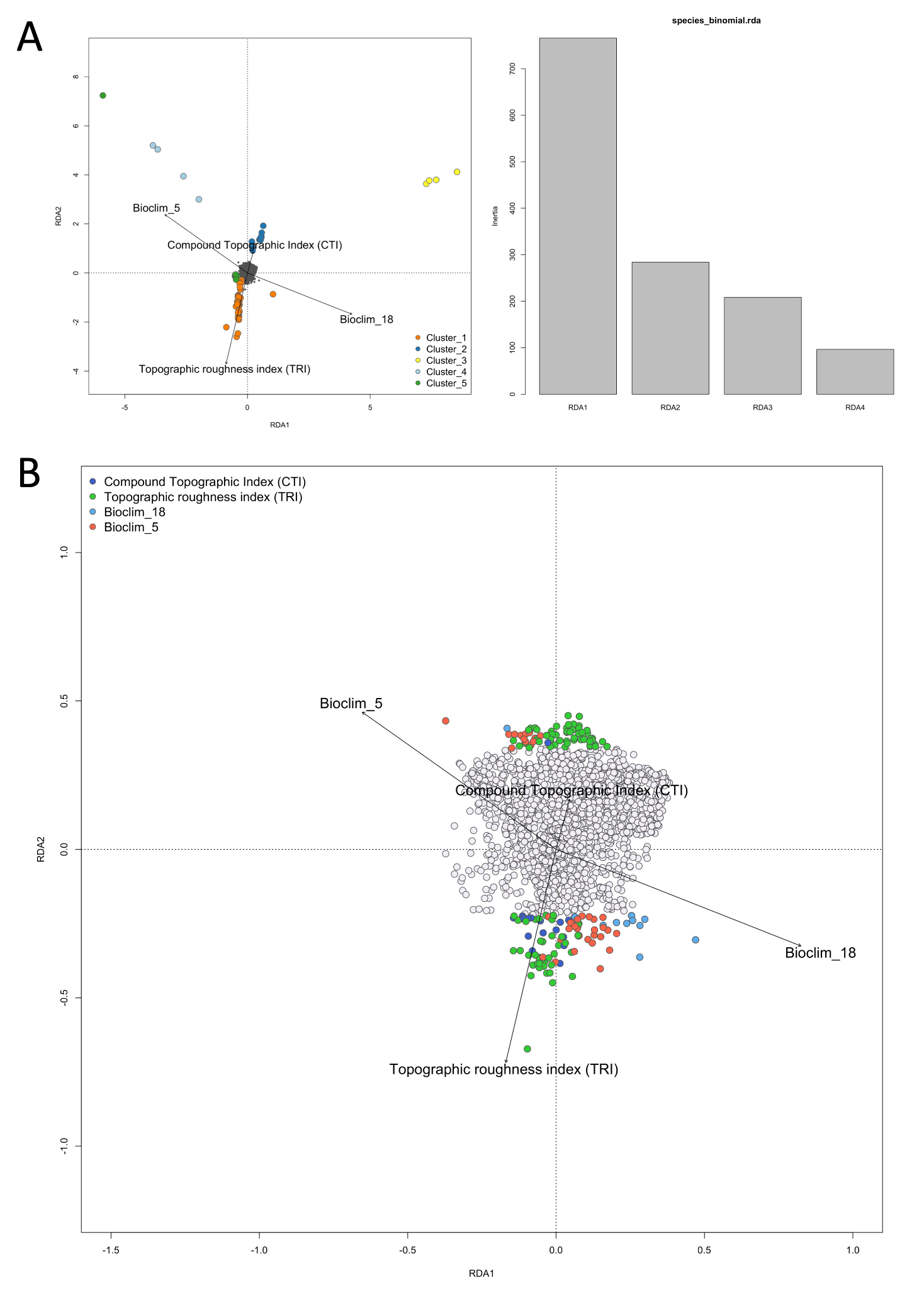


**Fig. S6.** A) RDA plot of population clusters in ordination space and their clustering relative to the environmental predictors used. k=6 is plotted to demonstrate that four populations is a reasonable assumption. B) SNPs identified as potentially under selection with a SD>2.5 from the mean loadings of the RDA relative to the environmental predictors.


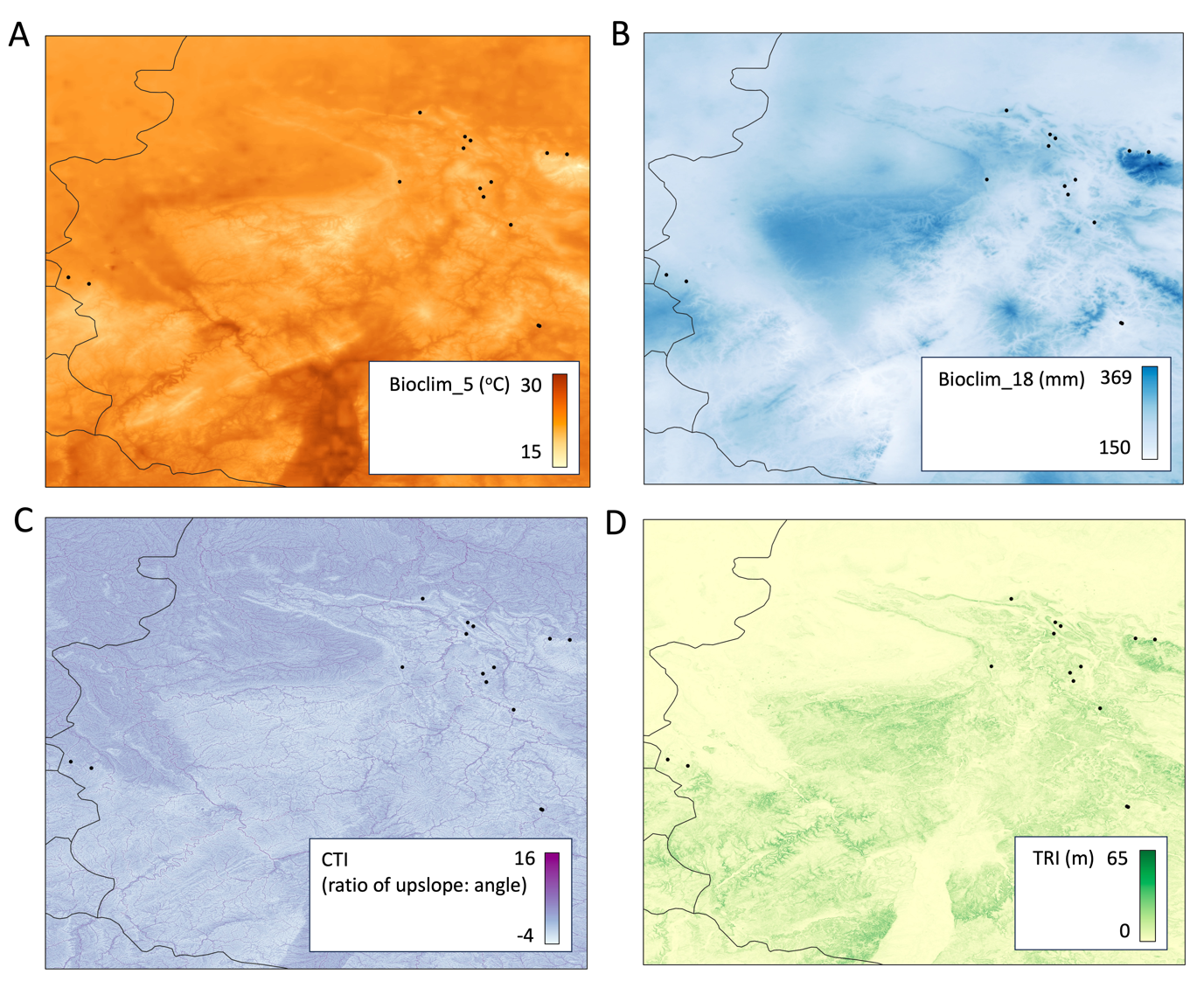


**Fig. S7.** Spatial variation of the four predictors used in our Genotype-Environment Association Analyses. Sampling localities for *Alytes obstetricans* are represented by black dots. A) Maximum temperature of the warmest month (Bioclim_5), measured in ^o^C. B) Precipitation of the warmest quarter (Bioclim_18), measured in mm. C) Compound Topographic Index (CTI), measured as a ratio of upslope to slope angle). D) Terrain Roughness Index (TRI), measured as the change in metres elevation per pixel.

**Table S1**. Pairwise genetic differentiation (F_ST_) between sampling localities calculated using Microsatellite and ddRAD-seq data. F_ST_ calculated using the approach of Weir and Cockerham (1984) to account for uneven sampling with an alpha significance level of 0.05 based on 100 bootstrap replicates.


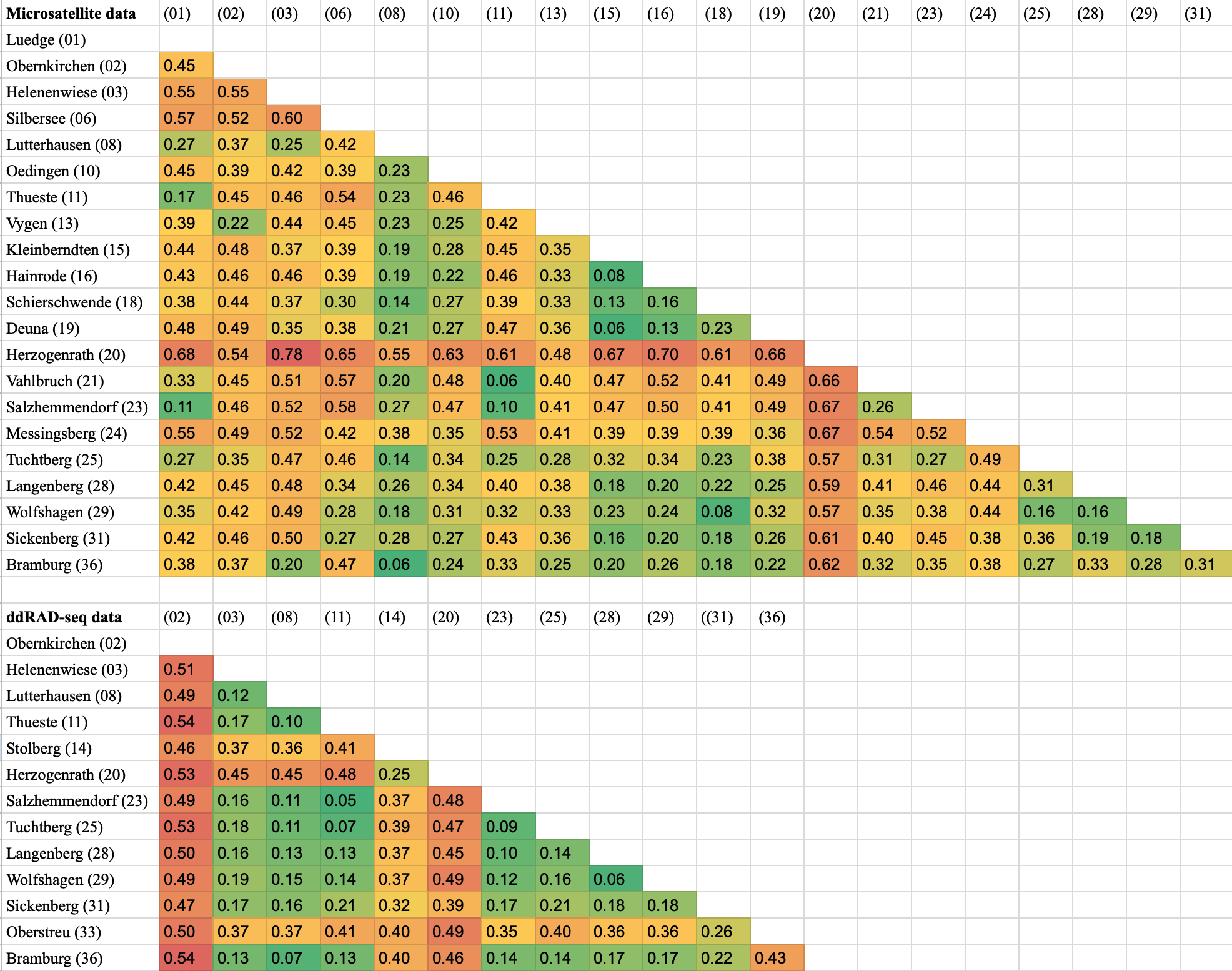


**Table S2**. Genetic diversity estimates from ddRAD-seq data using both fixed and variant sites.

| **Sampling locality** | **Latitude** | **Longitude** | **PA** | **Ho** | **He** |
| --- | --- | --- | --- | --- | --- |
| Tuchtberg (25) | 51.954653 | 9.592075 | 966 | 0.00161 | 0.00095 |
| Thueste (11) | 52.021861 | 9.653794 | 1146 | 0.00171 | 0.00104 |
| Wolfshagen (29) | 51.91185 | 10.329069 | 2291 | 0.0016 | 0.00085 |
| Salzhemmendorf (23) | 52.055406 | 9.604947 | 4403 | 0.0012 | 0.00062 |
| Stolberg (14) | 50.77247 | 6.29052 | 1970 | 0.00173 | 0.00106 |
| Helenenwiese (03) | 51.530744 | 9.768711 | 665 | 0.00155 | 0.00092 |
| Lutterhausen (08) | 51.660211 | 9.835944 | 299 | 0.00141 | 0.00078 |
| Obernkirchen (02) | 52.265169 | 9.208136 | 5020 | 0.00156 | 0.00091 |
| Oberstreu (33) | 50.410436 | 10.251206 | 8439 | 0.00116 | 0.00074 |
| Langenberg (28) | 51.901783 | 10.504325 | 1888 | 0.00154 | 0.00094 |
| Herzogenrath (20) | 50.82914 | 6.10896 | 3478 | 0.00172 | 0.00107 |
| Sickenberg (31) | 51.286983 | 10.009067 | 1878 | 0.00171 | 0.00108 |
| Bramburg (36) | 51.604287 | 9.738438 | 1661 | 0.00171 | 0.00113 |
| Boneburg (37) | 51.662 | 9.0289 | 3138 | 0.00111 | 0.00056 |

**References**

Gilbert, M. T. P., Haselkorn, T., Bunce, M., Sanchez, J. J., Lucas, S. B., Jewell, L. D., et al. (2007). The Isolation of Nucleic Acids from Fixed, Paraffin-Embedded Tissues–Which Methods Are Useful When? PLOS ONE, 2(6), e537. <https://doi.org/10.1371/journal.pone.0000537>

Maia-Carvalho, B., Gonçalves, H., Martínez-Solano, I., Gutiérrez-Rodríguez, J., Lopes, S., et al. (2014). Intraspecific genetic variation in the common midwife toad (*Alytes obstetricans*): Subspecies assignment using mitochondrial and microsatellite markers. *Journal of Zoological Systematics and Evolutionary Research*, *52*(2), 170–175. <https://doi.org/10.1111/jzs.12048>

Tobler, U., Garner, T. W. J., & Schmidt, B. R. (2013). Genetic attributes of midwife toad (*Alytes obstetricans*) populations do not correlate with degree of species decline. *Ecology and Evolution*, *3*(9), 2806–2819. <https://doi.org/10.1002/ece3.677>

Truong, H. T., Ramos, A. M., Yalcin, F., de Ruiter, M., van der Poel, H. J. A., et al. (2012) Sequence-Based Genotyping for Marker Discovery and Co-Dominant Scoring in Germplasm and Populations. PLOS ONE 7(5): e37565. https://doi.org/10.1371/journal.pone.0037565
